# Primary Atrial Leiomyosarcoma in a Patient with a Prior History of Uterine Leiomyoma and Ovarian Tumour

**DOI:** 10.1016/j.cjco.2025.03.008

**Published:** 2025-03-12

**Authors:** Solomon Tuonuur, Nasreen Shaikh, Radishma Kumar, Alireza Setarehaseman, Alessandra F. Nascimento, Hossein Akhondi, Abbas Mohammadi

**Affiliations:** a Department of Internal Medicine, Valley Health System, Las Vegas, Nevada, USA; b Department of Pathology, University Hospitals Cleveland Medical Center and Case Western Reserve University School of Medicine, Cleveland, Ohio, USA

**Primary cardiac leiomyosarcomas are exceedingly rare malignancies. They present insidiously with symptoms often attributable to mass effect or obstruction of blood flow, leading to delayed diagnosis. We present a rare case of primary cardiac leiomyosarcoma with a history of exophytic leiomyoma. Interesting to note is that this is a case of a primary cardiac tumour in a patient with a history of extracardiac malignancies. Histopathologic confirmation is crucial for timely diagnosis and appropriate management. Aggressive surgical resection remains the mainstay of treatment, but the high recurrence rates necessitate ongoing surveillance and consideration of adjuvant therapies.**

## Background

Primary cardiac tumours, originating from the heart's tissues, are exceedingly rare entities, accounting for < 0.2% of all cardiac neoplasms.^1^^,^^2^ Among these, leiomyosarcomas, malignant tumours arising from smooth muscle cells, constitute an even smaller subset.^2^ The clinical presentation of cardiac leiomyosarcoma is often insidious, with symptoms arising due to mass effect, obstruction of blood flow, or distant metastasis. Dyspnea, chest pain, palpitations, and syncope are common presenting complaints, often leading to delayed diagnosis, and advanced disease at presentation.^1^^,^^3^

The diagnostic workup typically involves a combination of imaging modalities, including echocardiography, computed tomography, and magnetic resonance imaging.^1^^,^^3^ These techniques aid in characterizing the tumour's size, location, and extent of involvement, and they guide subsequent management decisions. Definitive diagnosis, however, relies on histopathologic examination and immunohistochemical staining to differentiate leiomyosarcoma from other cardiac tumours.^1^^,^^2^^,^^4^

Surgical resection remains the cornerstone of treatment for primary cardiac leiomyosarcoma. Complete excision, whenever feasible, offers the best chance for long-term survival. However, the infiltrative nature of these tumours often poses technical challenges, and complete resection may not always be achievable.^1^^,^^2^^,^^4^ The high propensity for their recurrence necessitates vigilant surveillance and consideration of adjuvant therapies, such as chemotherapy and radiation therapy, although their role remains controversial due to limited evidence.

The prognosis for patients with primary cardiac leiomyosarcoma is generally poor, with reported survival in most patients in the range of 6-24 months. Factors influencing prognosis include tumour size, tumour location, resection completeness, and metastasis.^5^

## Case Presentation

A 39-year-old female patient with a past medical history significant for endometriosis, exophytic leiomyoma treated with myomectomy, and a borderline left mucinous ovarian tumour managed with salpingo-oophorectomy presented to the emergency department with a 14-day history of progressive dyspnea and intermittent chest pressure. The dyspnea initially occurred only with exertion, but it rapidly progressed to occurring at rest, interfering with her sleep. She denied any other associated symptoms, including chest pain, palpitations, cough, or lower-extremity edema.

**Figure 1:**
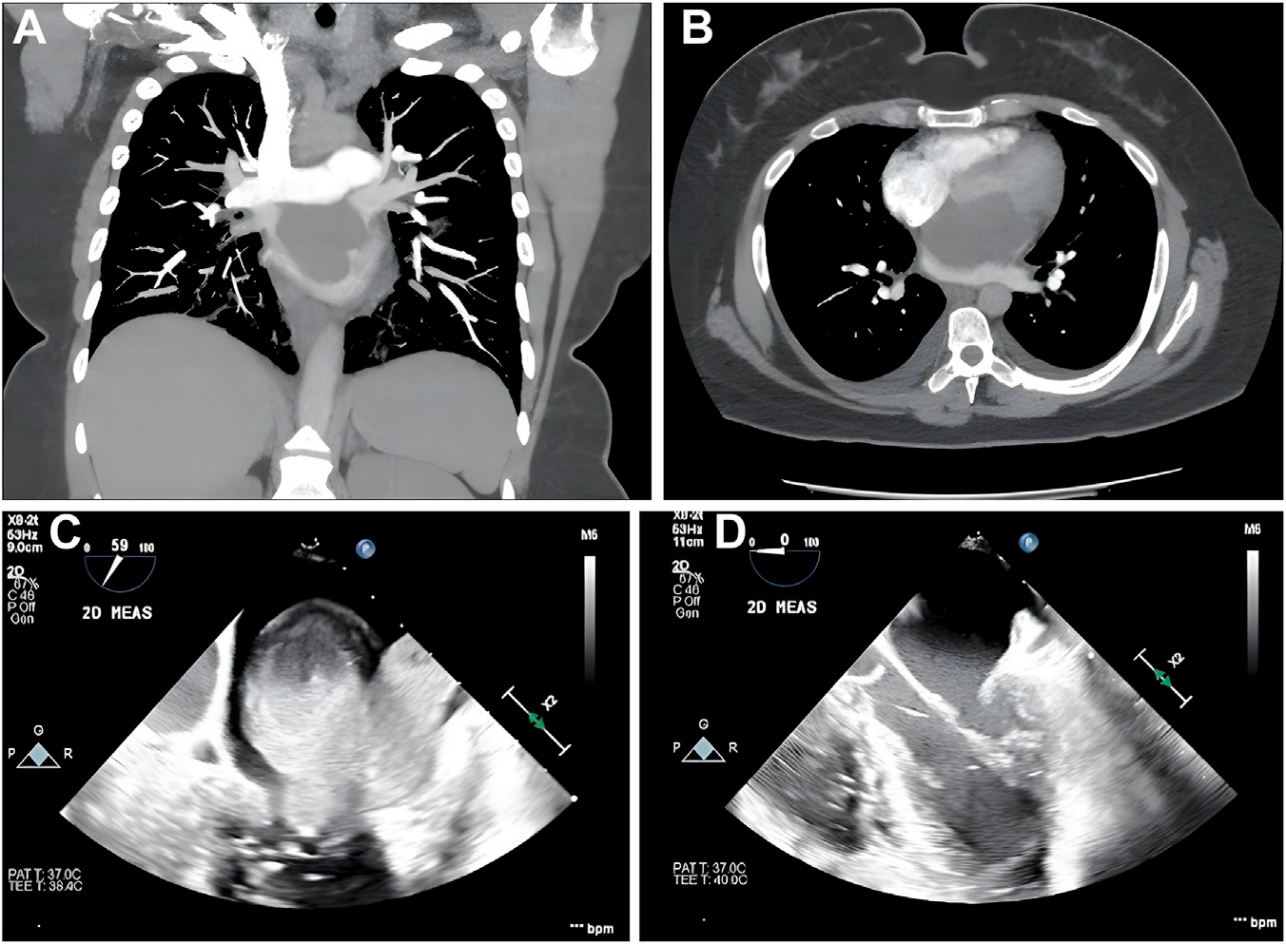
Imaging findings before and after surgery. (**A**) Computed tomography angiogram: coronal view showing a large, lobulated mass located in the left atrium of the heart. (**B**) Computed tomography angiogram (axial view): The mass extends through the mitral valve into the left ventricle. (**C**) Preoperative transesophageal echocardiogram: a 5.6 x 5.8 cm tumour with broad attachment to the posterior wall of the left atrium. (**D**) Postoperative transesophageal echocardiogram: clear left atrium and ventricle following tumour resection.

Physical examination revealed tachycardia and obesity (body mass index, 35), with no murmurs, crackles, or edema. Laboratory findings showed elevated white blood cell count (12,160), D-dimer (1.46), and pro-B-type natriuretic peptide level (536). Chest X ray demonstrated a typical cardiac silhouette with peri-hilar infiltrates. A computed tomography angiogram revealed a large left atrial mass (6.8 x 4.8 cm) extending through the mitral valve into the left ventricle (Fig. 1, A and B). A transesophageal echocardiogram confirmed a 5.6 x 5.8 cm mass attached to the posterior wall, with severe mitral regurgitation and preserved left ventricular ejection fraction (60%-65%; Fig. 1C). The tumour was excised surgically (Fig. 1D) through a median sternotomy, with the procedure supported by cardiopulmonary bypass. A left atriotomy was performed to remove a 7-cm left atrial mass, which completely filled the cavity and exhibited fibrous characteristics. The mass was adherent to the aorto-mitral curtain, the anterior mitral leaflet, and the interatrial septum. Using meticulous dissection techniques, the mass was excised intact, with no visible residual tissue left on the atrial wall, septum, or adjacent structures. The cross-clamp time was 44 minutes, and the total bypass time was 55 minutes. Intraoperative examination confirmed the mitral valve's competence, with no evidence of mitral regurgitation. Margins were thoroughly assessed, and no residual tumour was identified. The left atrial appendage was excluded using an AtriCure clip (AtriCure, Inc, BIBA Medical, Way Mason, OH). After closure of the left atrium, the patient was weaned off cardiopulmonary bypass, and her ventricular function was preserved, with an ejection fraction of 55%. The surgical site was closed, and the patient was transferred to the intensive care unit in stable condition.

Gross examination of the resected specimen showed a tan to gray-white, smooth, and glistening mass measuring 7.5 x 5.5 x 4.2 cm, with a focally polypoid appearance. One surface was ragged, possibly indicating the resection margin. Cut surfaces revealed tan-gray tissue with focal pale red and/or yellow discoloration.

**Figure 2:**
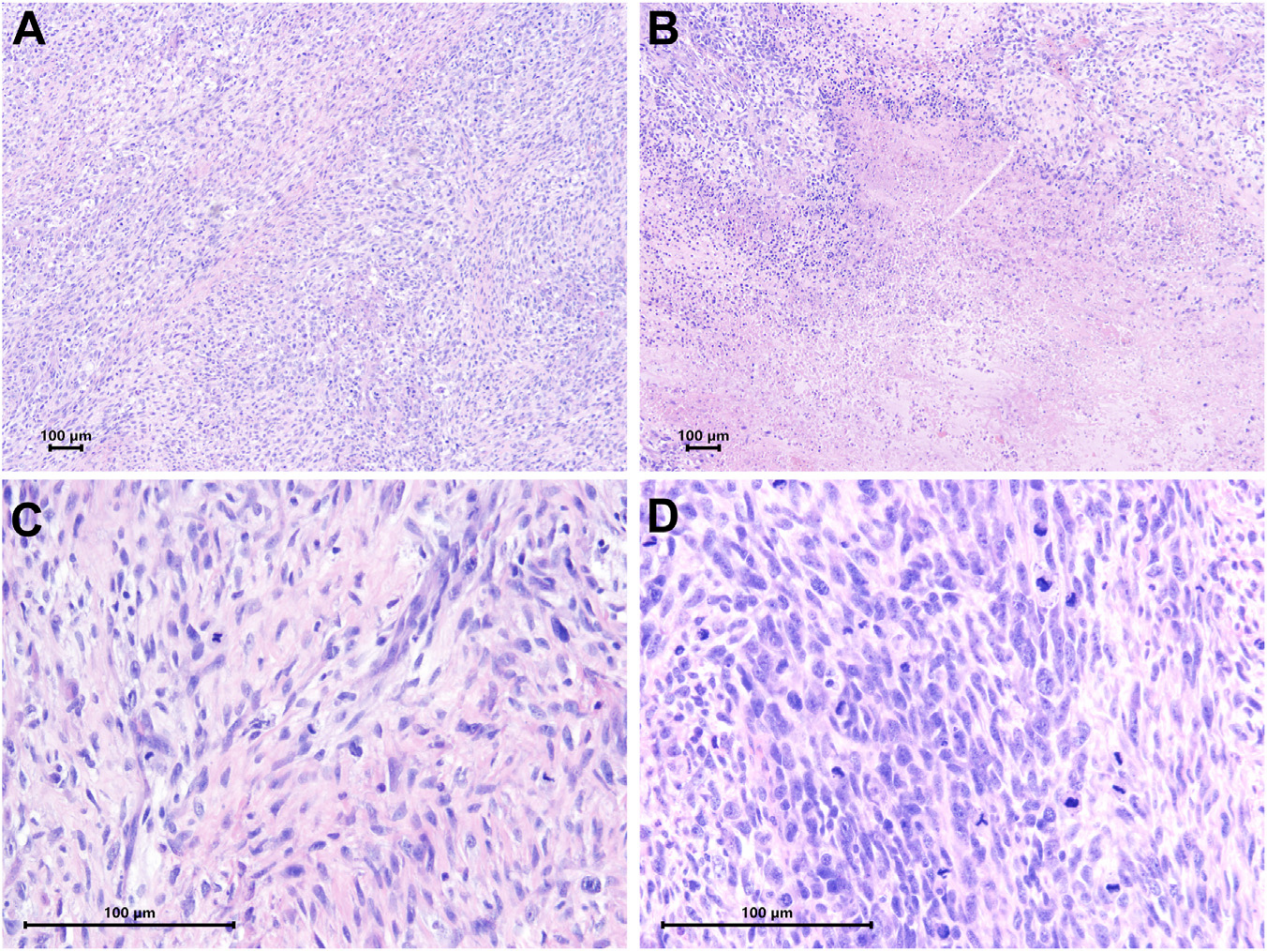
Histopathologic slides. Hematoxylin and eosin stain shows (**A**) a proliferation of spindle cells with (**B**) areas of necrosis. (**C, D**) Higher magnification demonstrates severe nuclear atypia with high mitotic activity.

Microscopic examination demonstrated a proliferation of spindle cells with moderate-to-severe nuclear atypia, areas of necrosis, and a high mitotic rate (19 mitoses per 10 high-power fields; Fig. 2, A-D). Immunohistochemical staining was strongly positive for smooth muscle markers smooth muscle actin and desmin, confirming the tumour's smooth muscle origin (Supplemental Figs. S1 and S2). The tumour cells were negative for neural markers (S100, SOX10), endothelial markers (CD34), and epithelial markers (AE1/AE3). Additionally, the tumour was negative for estrogen receptor and progesterone receptor, ruling out a potential metastatic spread from a uterine source. A previous hysterectomy specimen was reviewed, and this confirmed the diagnosis of leiomyoma, further supporting the primary cardiac origin of the current tumour.

The patient was discharged in a stable condition and referred to an oncology centre for adjuvant chemotherapy.

## Discussion

Primary cardiac leiomyosarcomas, originating from smooth muscle cells within the heart, are exceptionally rare malignancies (0.025% of all sarcomas),^1^ with the left atrium being their most common location.^6^ We present the case of a patient with a left atrial leiomyosarcoma who remained asymptomatic until the tumour caused mass effect symptoms, highlighting the potential for delayed presentation.

These tumours often have an insidious clinical presentation, with symptoms arising from mass effect or obstruction of blood flow. Dyspnea, as seen in our patient, is a common presenting complaint, often accompanied by chest pain, palpitations, or syncope. Despite the patient's prior history of uterine leiomyoma, no histochemical link was established with the atrial leiomyosarcoma, and metastasis from the uterus was unlikely, given that most uterine leiomyosarcomas spread to the lungs.^1^^,^^3^^,^^5^

Diagnosis typically involves a combination of imaging modalities, including echocardiography, computed tomography, and magnetic resonance imaging to characterize the tumour's size, location, and extent. Definitive diagnosis relies on histopathologic examination and immunohistochemical staining.

Surgical resection remains the cornerstone of treatment for primary cardiac leiomyosarcomas. Complete excision, whenever feasible, offers the best chance for long-term survival.^5^ However, the infiltrative nature of these tumours often makes complete resection challenging. The high propensity for recurrence necessitates vigilant surveillance and consideration of adjuvant therapies.

The role of adjuvant chemotherapy and radiation therapy remains controversial, with limited evidence to support their routine use. Research is ongoing to determine the optimal chemotherapeutic regimen and timing of administration.^6^^,^^7^

Factors influencing prognosis include tumour size, tumour location, resection completeness, and the presence of metastasis. Although our patient had no evidence of metastasis at diagnosis, the tumour exhibited high mitotic activity and necrosis, which may portend a less-favourable outcome. Overall survival for primary cardiac leiomyosarcoma is 24 months after resection, and 6-10 months without resection.^5^

This case of a primary cardiac leiomyosarcoma serves as a poignant reminder of the challenges involved in diagnosing and managing these rare tumours. The case highlights the crucial need for a high index of suspicion, even when patients present with seemingly unrelated symptoms or have a history of extracardiac malignancies. Only through a comprehensive approach, encompassing thorough clinical assessment, advanced imaging techniques, and expert histopathologic analysis, can we ensure timely and accurate diagnosis. In addition, optimal management necessitates a multidisciplinary collaboration among cardiologists, oncologists, and surgeons, working together to devise individualized treatment strategies and improve outcomes for this complex patient population.

**Novel Teaching Points**

• This case demonstrates a rare instance of primary cardiac leiomyosarcoma in a patient with a history of exophytic leiomyoma. Immunohistochemical analysis confirmed the tumour’s negativity for estrogen receptor and progesterone receptor, ruling out metastatic spread from a uterine source and establishing this as a primary malignancy.
• The case highlights the importance of not anchoring diagnoses based solely on a patient's prior history of cancer. A thorough and detailed investigation is crucial for any new presentation, even when the symptoms may seem unrelated to the previous malignancy.
• Early surgical resection is crucial for primary cardiac leiomyosarcomas, but the tumour's rarity and infiltrative nature emphasize the need for careful consideration of adjuvant therapies and ongoing surveillance for recurrence, despite the lack of oncology follow-up in this case.
