# Supplementary material for: Primary Atrial Leiomyosarcoma in a Patient with a Prior History of Uterine Leiomyoma and Ovarian Tumour
Source: CJC Open. 2025 Mar 12;7(5):671–4. doi: 10.1016/j.cjco.2025.03.008 (PMC12105520; doi:10.1016/j.cjco.2025.03.008)

Supplemental Figure S1: Immunohistochemical staining. Strong positivity for smooth muscle markers desmin.

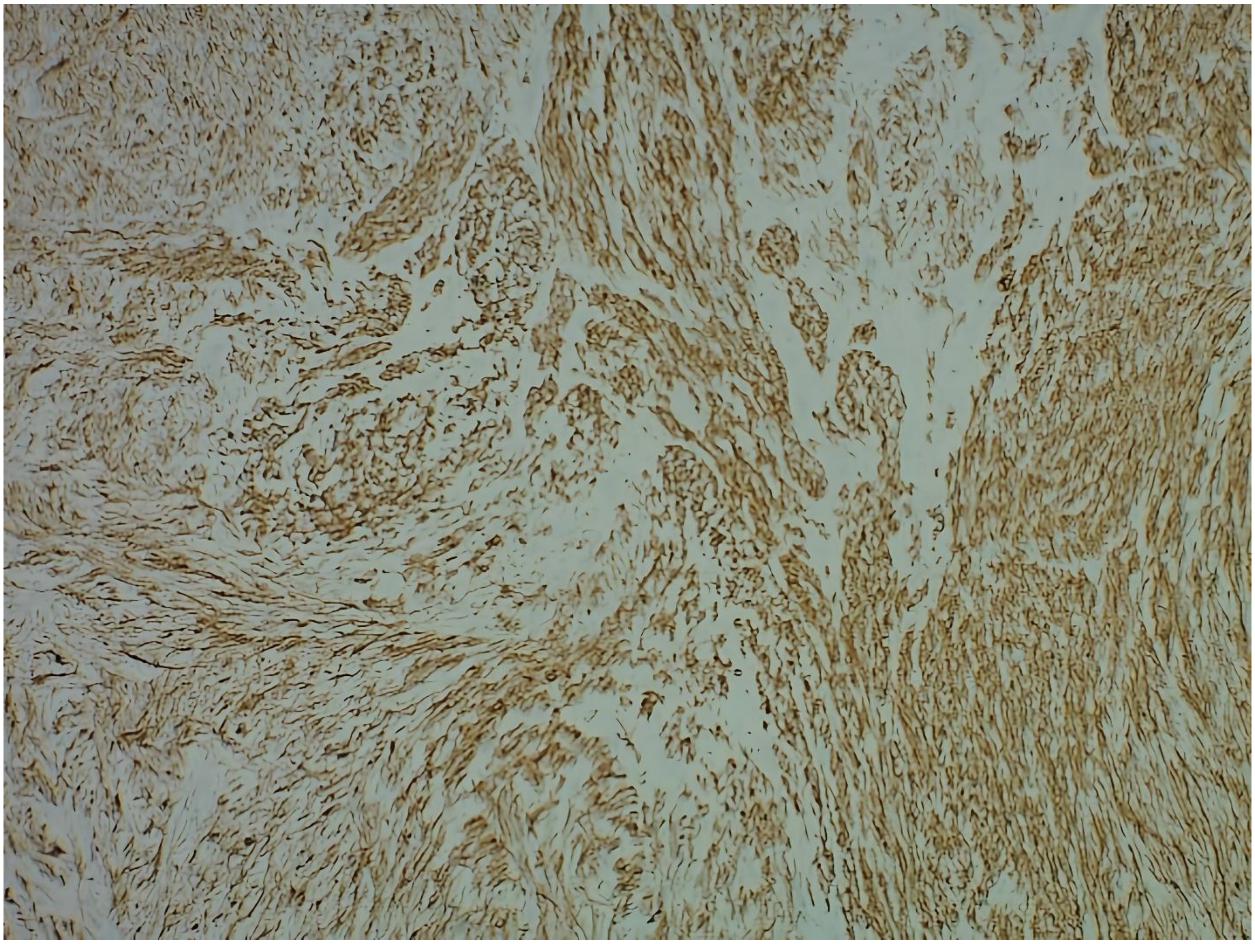

Supplemental Figure S2: Immunohistochemical staining. Strong positivity for smooth muscle actin (SMA).

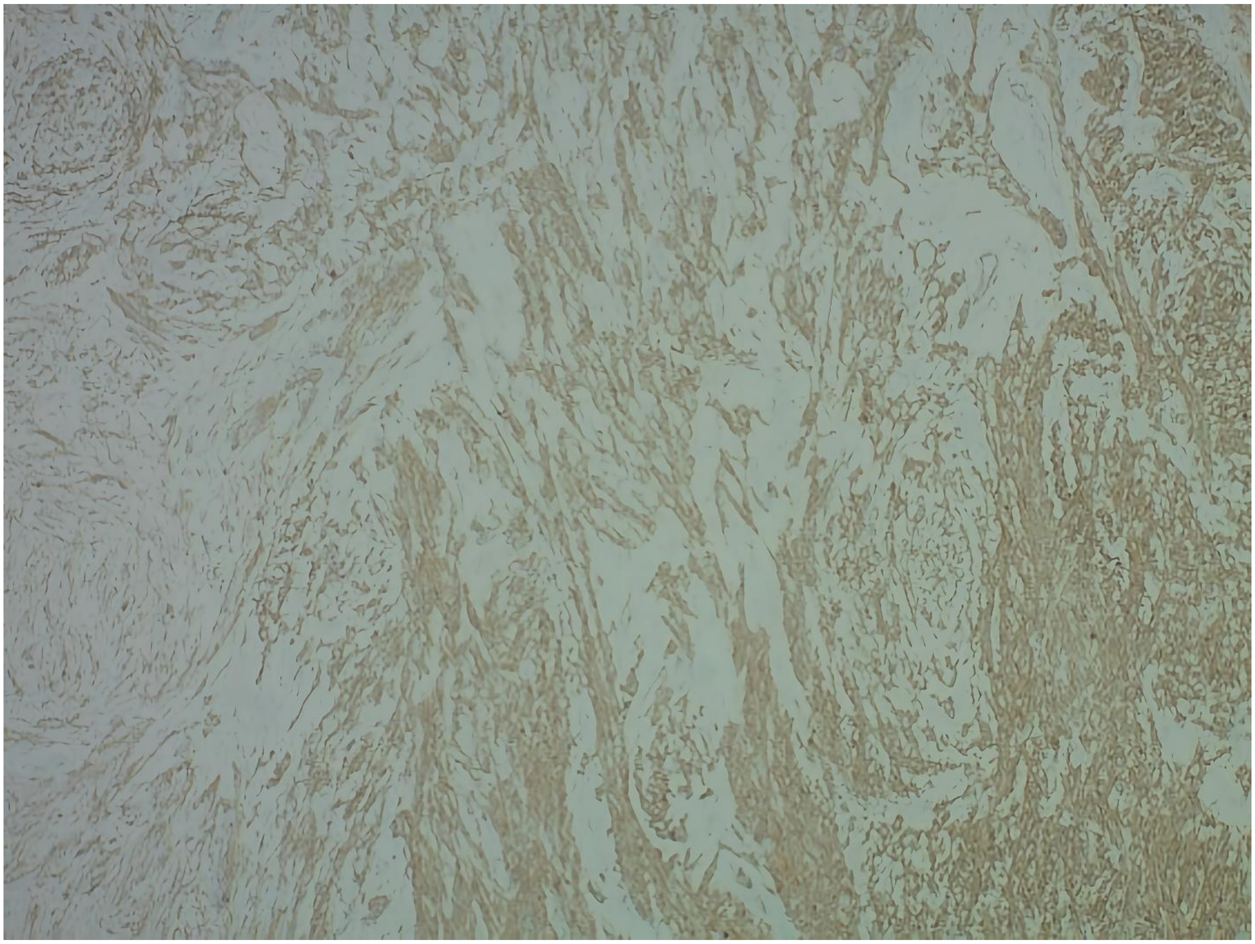

Supplement: Supplemental Figure [file mmc1.pdf]
